# Pharmacological and Non-Pharmacological Treatments for Depression in Parkinson’s Disease: An Updated Review

**DOI:** 10.3390/medicina59081454

**Published:** 2023-08-12

**Authors:** Efthalia Angelopoulou, Evangelia Stanitsa, Claire Chrysanthi Karpodini, Anastasia Bougea, Dionysia Kontaxopoulou, Stella Fragkiadaki, Christos Koros, Vasiliki Epameinondas Georgakopoulou, George Fotakopoulos, Yiannis Koutedakis, Christina Piperi, Sokratis G. Papageorgiou

**Affiliations:** 1 1st Department of Neurology, Medical School, National and Kapodistrian University of Athens, Eginition Hospital, 11528 Athens, Greece; angelthal@med.uoa.gr (E.A.); eva.st.92@gmail.com (E.S.); annita139@yahoo.gr (A.B.); d.kontaxopoulou@hotmail.com (D.K.); st.fragkiadaki@gmail.com (S.F.); chkoros@gmail.com (C.K.); 2 Department of Biological Chemistry, Medical School, National and Kapodistrian University of Athens, 11527 Athens, Greece; cpiperi@med.uoa.gr; 3 Sport and Physical Activity Research Centre, Faculty of Education, Health and Wellbeing, University of Wolverhampton, Wolverhampton WV1 1LY, UK; claire_karpodini@outlook.com; 4 Department of Infectious Diseases-COVID-19 Unit, Laiko General Hospital, 11527 Athens, Greece; vaso_georgakopoulou@hotmail.com; 5 Department of Neurosurgery, General University Hospital of Larissa, 41221 Larissa, Greece; gfotakop@yahoo.gr; 6 Functional Architecture of Mammals in Their Environment Laboratory, Department of Physical Education and Sport Science, University of Thessaly, 38221 Volos, Greece; y.koutedakis@gmail.com

**Keywords:** Parkinson’s disease, depression, SSRIs, SNRIs, TCAs, MAO-B inhibitors, non-pharmacological, complementary, CBT, physical exercise

## Abstract

Depression represents one of the most common non-motor disorders in Parkinson’s disease (PD) and it has been related to worse life quality, higher levels of disability, and cognitive impairment, thereby majorly affecting not only the patients but also their caregivers. Available pharmacological therapeutic options for depression in PD mainly include selective serotonin reuptake inhibitors, serotonin and norepinephrine reuptake inhibitors, and tricyclic antidepressants; meanwhile, agents acting on dopaminergic pathways used for motor symptoms, such as levodopa, dopaminergic agonists, and monoamine oxidase B (MAO-B) inhibitors, may also provide beneficial antidepressant effects. Recently, there is a growing interest in non-pharmacological interventions, including cognitive behavioral therapy; physical exercise, including dance and mind–body exercises, such as yoga, tai chi, and qigong; acupuncture; therapeutic massage; music therapy; active therapy; repetitive transcranial magnetic stimulation (rTMS); and electroconvulsive therapy (ECT) for refractory cases. However, the optimal treatment approach for PD depression is uncertain, its management may be challenging, and definite guidelines are also lacking. It is still unclear which of these interventions is the most appropriate and for which PD stage under which circumstances. Herein, we aim to provide an updated comprehensive review of both pharmacological and non-pharmacological treatments for depression in PD, focusing on recent clinical trials, systematic reviews, and meta-analyses. Finally, we discuss the pharmacological agents that are currently under investigation at a clinical level, as well as future approaches based on the pathophysiological mechanisms underlying the onset of depression in PD.

## 1. Introduction

Neurodegenerative diseases cause a substantial health burden worldwide and their prevalence is on the rise. After Alzheimer’s disease (AD), Parkinson’s disease (PD) is the second most frequent neurodegenerative disorder, affecting more than 1% of the elderly population [1]. The exact pathogenesis of PD remains obscure; although, several genetic factors and environmental exposures, as well as a complex interaction between them, contribute to its development [2,3]. Concerning the underlying mechanisms, mitochondrial dysfunction, autophagy dysregulation, neuroinflammation, oxidative stress, and proteasome impairment have been shown to play a pivotal role [2,4]. The neuropathological hallmarks of PD are the progressive loss of dopaminergic neuronal cells in the substantia nigra pars compacta (SNpc) in the midbrain and the accumulation of Lewy bodies and Lewy neurites, which primarily consist of alpha-synuclein protein aggregates [2]. The cardinal motor features of PD include resting tremors, rigidity, bradykinesia, and postural instability while most PD patients also experience non-motor symptoms, such as sleep disorders, autonomic dysfunction, cognitive decline, psychotic manifestations, anxiety, and depression [5]. Non-motor symptoms may actually be more burdensome compared to motor ones and, although levodopa still represents “the gold standard” approach for the symptomatic treatment of PD motor symptoms, the management of non-motor manifestations is especially challenging [6].

Depression represents one of the most common non-motor disorders in PD, affecting approximately 30–40% of PD patients; although, epidemiological evidence across different studies varies widely [7]. In PD, depression has been associated with reduced quality of life, worse levels of disability, cognitive impairment, and increased mortality and morbidity, thereby majorly affecting both patients and their caregivers [6]. Depression appears in all PD stages and it may precede the onset of motor impairment during the prodromal stages of the disease [6]. Risk factors for depression in PD include female sex, increased physical dependence, higher levodopa dose, more severe motor impairment, pain, and daytime sleepiness [8]. Clinically, PD patients with depression display less prominent guilt and self-hate compared to depressed non-PD individuals; meanwhile, the core symptoms of loss of satisfaction, enjoyment/pleasure, and appetite commonly occur [6]. The underlying pathophysiological mechanisms of PD depression are complex, involving impaired cortico-striatal and limbic brain circuits, an imbalance between neurotransmitter systems (mainly serotoninergic, dopaminergic, and noradrenergic), neuroinflammation, and the dysregulation of neurotrophic factors [8].

Depression in PD might go unrecognized since some symptoms, including weight loss, sleep disturbances, psychomotor slowing, and fatigue, can be attributed to the disease itself, regardless of the co-occurrence of depression [9,10]. The early diagnosis and effective treatment of PD depression are of paramount importance and involve both pharmacological and non-pharmacological therapeutic interventions. However, evidence of the effectiveness and safety of antidepressant medications used against depression in PD is inconsistent, the optimal treatment approach is uncertain, and its management may be challenging [11]. Definite guidelines for PD depression are also lacking [12]. Furthermore, although several non-pharmacological interventions have shown promising potential in the management of PD depressive symptoms, it is still unclear which of these interventions are the most appropriate and for which PD stage under which circumstances. Currently, dual serotonin and noradrenaline reuptake inhibitors (SNRIs), such as nortriptyline and venlafaxine, and selective serotonin and reuptake inhibitors (SSRIs), such as fluoxetine and citalopram, are generally firstly recommended, followed by tricyclic antidepressants (TCAs) [12]. In addition, physical exercise; psychotherapy mainly in the form of cognitive behavioral therapy (CBT); and repetitive transcranial magnetic stimulation (rTMS) can also be considered, despite the inadequately robust evidence [6,11,13].

Herein, given the growing number of studies focusing on the treatment of PD depression, we aim to provide an updated comprehensive review of the existing literature about the extensive range of both pharmacological and non-pharmacological treatments for depression in PD, focusing on recent clinical trials, systematic reviews, and meta-analyses. In addition, we discuss the pharmacological agents that are currently under investigation at a clinical level, together with future approaches based on the pathophysiological mechanisms underlying the development of depression in PD.

For this purpose, we searched PubMed and Scopus databases for peer-reviewed research and review articles investigating and/or discussing the pharmacological and non-pharmacological treatments of PD depression, published in the English language with no time restrictions. The search was conducted between September 2022 and April 2023. Both pre-clinical and clinical studies, as well as narrative and systematic reviews and meta-analyses, were included. In order to include most of the key recent evidence, we screened the references of the selected articles for additional relevant articles. We used the terms “Parkinson’s disease”, “depressive”, “depression”, “psychiatric”, “neuropsychiatric”, “mood”, “treatment”, “therapy”, “pharmacological”, “non-pharmacological”, “antidepressants”, “levodopa”, “dopaminergic”, “monoamine oxidase”, “exercise”, “training”, and “psychotherapy” in different combinations. For the narrative synthesis, our initial categorization was based on the various pharmacological and non-pharmacological treatment approaches having been clinically investigated in PD depression, followed by a discussion on potential future directions based on the results of the relevant studies.

## 2. Pharmacological Treatment Approaches for Depression in PD

**Figure 1:**
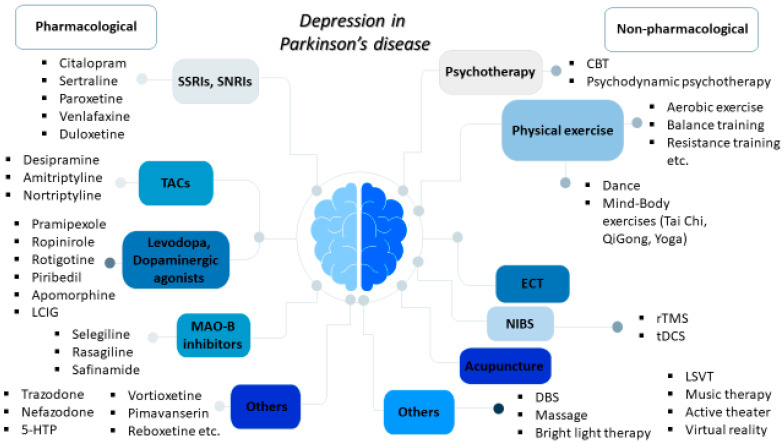
Overview of the pharmacological and non-pharmacological treatments for depression in Parkinson’s disease.

The use of antidepressant medications, including SSRIs, SNRIs, and TCAs, is the most common treatment strategy for PD depression [14]. It is estimated that up to 25% of PD patients may be on antidepressants at anytime during the course of the disease [15] and SSRIs are more frequently prescribed compared to TCAs [16]. Agents acting on dopaminergic pathways, such as levodopa, dopaminergic agonists, and monoamine oxidase B (MAO-B) inhibitors, may also display antidepressant effects. In the following subsections, we discuss recent evidence of the role of pharmacological interventions currently used in PD depression, as well as the potential effects of other drugs that are currently under clinical investigation (Figure 1 and Table 1).

### 2.1. Tricyclic Antidepressants, Selective Serotonin Reuptake Inhibitors, and Serotonin and Norepinephrine Reuptake Inhibitors 

In addition to the degeneration of SNpc in PD, there is also a loss of serotoninergic neurons in the raphe nucleus. PD patients with depression display reduced serotonin levels; whereas, serotonin binding is higher in specific brain regions compared to PD patients not suffering from depression [8]. Along with the dysregulation of the serotoninergic system, degeneration of the locus coeruleus, the main brain area producing norepinephrine, is also observed in PD. The lack of noradrenergic stimulation in this region and the thalamus is also linked to PD depression [8].

TCAs act by inhibiting the reuptake of norepinephrine and serotonin; they have been demonstrated to be effective in treating depression in PD patients. However, TCAs may be associated with several side effects, including orthostatic hypotension; sedation; and anticholinergic adverse effects, such as confusion and impaired concentration, which may be problematic for PD patients [17]. Of note, the use of TCAs should be avoided in patients with narrow angle-closure glaucoma and cardiac abnormalities, such as left bundle-branch block, QTc interval prolongation, or ischemic heart disease [6,12]. A randomized placebo-controlled clinical trial has demonstrated that desipramine, a primarily noradrenergic TCA, could significantly improve depressive symptoms in PD patients compared to a placebo [18]. In this clinical trial, the efficacy of desipramine was better compared to that of citalopram, a SSRI, on the 14th day of treatment; but, their effectiveness was similar after 30 days, suggesting that desipramine provides a finally equal but slightly earlier therapeutic benefit. However, mild adverse effects were twice as common with desipramine than with citalopram or a placebo, highlighting its lower tolerability. Clinical trials have also indicated that amitriptyline may also improve depressive symptoms in PD [19,20]. Nortriptyline, another TCA, was also more effective for PD depression in another randomized placebo-controlled trial within 8 weeks of treatment compared to paroxetine, a SSRI, or a placebo [21].

SSRIs display generally similar effectiveness and they are more well-tolerated compared to TCAs in non-PD elderly patients with depression [22,23]. The most frequent side effects of SSRIs include gastrointestinal abnormalities like nausea, as well as reduced libido, action tremor, and occasionally hyponatremia [12]. In addition, it has been reported that SSRIs may exacerbate motor parkinsonian symptoms [24,25]. As abovementioned, citalopram has been proven to be effective in treating PD depression in a clinical trial within 4 weeks compared to a placebo [18]. Sertraline also significantly improved depressive symptoms in PD in another randomized trial [19]. Paroxetine provided additional beneficial effects on PD depression in one more placebo-controlled trial, on the 12th week, without deteriorating motor symptoms [26]; whereas, no significant results were reported within 8 weeks in another placebo-controlled trial [21]. The shorter treatment duration and the increased dropout rate in the latter study may possibly explain these contradictory results [26].

SNRIs inhibit both serotonin and norepinephrine reuptake while displaying better tolerability than TCAs. Venlafaxine extended-release (XR), a SNRI, significantly improved depressive symptomatology compared to a placebo in patients with PD in a large multicenter randomized trial, without affecting motor function [26]. Another randomized open-label clinical trial has demonstrated that duloxetine, another SNRI, could improve depression in PD patients, in the 10th week, to a similar degree as SSRIs (paroxetine or escitalopram) [27].

Meta-analyses on the role of antidepressants in PD depression have demonstrated inconsistent results [12]. There is evidence showing that SSRIs do not provide significant benefits in PD depression compared to placebos (response rate 34% versus 36%), attributed to the considerably high placebo effect that is generally observed in clinical trials with antidepressants [12,15]. In agreement with these findings, another two meta-analyses confirmed the statistically insignificant efficacy of SSRIs and proposed that TCAs are more effective than SSRIs for PD depression [28,29]. The small sample sizes of the studies resulting in Type II errors could explain the lack of significance granted by the authors [29]. On the contrary, SSRIs significantly improved depression in PD in another meta-analysis while TCAs could not [30]. Both SSRIs and SNRIs were also found to be effective in another study [31] and two more recent meta-analyses demonstrated that both SSRIs and TCAs had significant effects on PD depression [32,33]. Regarding their side effects in PD patients, SSRIs and TCAs did not show statistically significant differences in terms of insomnia in another meta-analysis; but, xerostomia and constipation were more common with TCAs [34]. The different inclusion criteria and statistical methods, the high placebo effect, the small sample sizes, and the diverse instruments used for depression diagnosis could explain these conflicting results.

In conclusion, although definite recommendations cannot be proposed, it has been suggested that first-choice agents for PD depression are citalopram, venlafaxine, and paroxetine, followed by TCAs, given that there are no relative contraindications [12].

### 2.2. Levodopa and Dopaminergic Agonists

Dopaminergic neurotransmission has a major impact on the pathophysiology of depression in PD. The loss of dopaminergic neurons in the SNpc leads to reduced stimulation and dopamine release into the ventral striatum, which is highly implicated in motivation and reward regulation [8]. Furthermore, reduced dopamine levels are observed in the striatal regions of PD patients with depression compared to those without depression [8].

The critical first step for the medical care of PD patients is the optimal treatment of motor symptoms with dopaminergic therapy, mainly levodopa and dopaminergic agonists [6]. More precisely, in PD patients with motor fluctuations, it should be determined if the depressive symptomatology is closely related to the “off” phases since dopaminergic treatment modifications are necessary and likely more effective than the initiation of antidepressant pharmacotherapy in these cases [35]. Interestingly, it has also been demonstrated that dopamine replacement therapy, including levodopa and pramipexole, a dopaminergic agonist, may also alleviate depressive symptoms in PD [36,37]. Two meta-analyses confirmed these findings for PD patients in regard to depressive mood [38,39]. A following large randomized controlled trial indicated that pramipexole could probably exert direct antidepressant effects in PD compared to a placebo [37]. Furthermore, PD patients treated with pramipexole displayed greater recovery rates compared to sertraline in another clinical trial [40], suggesting that this dopaminergic agonist may be a favorable choice for PD patients suffering from depression. Ropinirole, one more dopaminergic agonist, could also improve depressive symptoms in PD, including those with levodopa-induced motor complications (dyskinesias, fluctuations) [41,42]. Two meta-analyses illustrated that rotigotine, a dopaminergic agonist delivered as a transdermal patch, was also able to significantly ameliorate the neuropsychiatric symptoms of PD patients, including depression [43,44]. Moreover, the use of the dopaminergic agonist piribedil could provide beneficial effects for apathy and depressive symptoms after DBS in the STN [45]. Importantly, a recent meta-analysis indicated that dopamine agonists could significantly improve depression in PD compared to a placebo [33]. In addition, continuous subcutaneous apomorphine infusion (CAI) [46] and intraduodenal levodopa-carbidopa intestinal gel (LCIG) [47] were also able to improve depression in patients with PD, indicating that infusion dopaminergic therapies may also be beneficial for mood improvement in PD [48].

Therefore, dopaminergic agonists, apart from their benefits on motor function, seem to exert some beneficial effects on PD depression as well. These agents may be considered especially for PD patients without psychotic features or increased risks of impulse control disorders, recently diagnosed at the early stages of PD, and for those whose depressive symptoms appeared after a decrease in the dose of dopaminergic treatment or those who are closely related to the “off” phases of motor fluctuations [35].

### 2.3. Monoamine Oxidase B Inhibitors

Selective irreversible MAO-B inhibitors, such as selegiline and rasagiline, can also enhance dopaminergic neurotransmission by suppressing dopamine metabolism mediated by MAO-B, thereby improving motor symptoms in PD. MAO-B inhibitors are generally well-tolerated; but, caution is needed in patients who receive SSRIs because of the (although low) risk of serotoninergic syndrome [12]. Rasagiline can also improve non-motor abnormalities in PD [49] and the combined use of rasagiline and antidepressants may also alleviate depressive symptoms, without causing serotoninergic syndrome as an adverse effect [50]. However, another 12-week randomized placebo-controlled trial indicated no significant effectiveness of rasagiline in improving depression in PD patients [51].

These conflicting results might be explained by the different inclusion criteria since the last study included PD patients with at least moderate depression [51]. Hence, the severity of PD depression might affect the effectiveness of dopaminergic agonists, such as rasagiline; the inconsistency of the inclusion criteria of the studies represents another important issue, which limits the comparability of their findings.

Selegiline was also effective in improving depressive symptoms in PD in a recent meta-analysis [52]. A systematic review showed that safinamide, a selective reversible MAO-B inhibitor and modulator of voltage-sensitive calcium and sodium channels that also affect glutamatergic neurotransmission, may also be beneficial for depression in PD [53]. Importantly, a meta-analysis indicated that MAO-B inhibitors displayed the greatest efficacy for PD depression, compared to SSRIs and TCAs [32], and two more recent meta-analyses also confirmed the effectiveness of MAO-B inhibitors in PD depression [33,54], especially for patients at early PD stages [54]. In summary, MAO-B seems to provide some antidepressant effects for patients with PD, particularly in the early stages of the disease.

### 2.4. Other Pharmacological Agents under Clinical Investigation

Along with SSRIs, SNRIs, and TACs, other agents acting on the serotoninergic pathway have also been investigated for PD depression. In particular, the 4-week use of 5-hydroxytryptophan, which is the chemical precursor and intermediate metabolite of L-tryptophan in the biosynthesis of serotonin, has been shown to improve depression in PD compared to a placebo in a randomized controlled trial [55]. Trazodone, a serotonin antagonist and reuptake inhibitor (SARI), was able to ameliorate depressive symptoms in PD patients in another randomized controlled trial [56]. Importantly, a pilot randomized clinical trial has shown that nefazodone, an atypical antidepressant acting as a SARI that selectively inhibits 5-HT2 receptors, could improve both depressive and motor symptoms in PD patients [57]. The stimulation of 5-HT2 receptors in the basal ganglia has been considered to contribute to the SSRI-mediated parkinsonian motor side effects, possibly by suppressing the release of dopamine [57]. Hence, nefazodone can exert benefits on both motor and depressive symptoms. In addition, there is an ongoing clinical trial investigating the antidepressant effects of vortioxetine in PD (NCT04301492); vortioxetine belongs to the category of “multimodal antidepressants”, acting by regulating serotonergic receptors, blocking serotonin reuptake, and affecting the activity of other neurotransmitters, including dopamine, histamine, norepinephrine, and acetylcholine [58].

Pimavanserin, an atypical antipsychotic acting as an inverse, selective agonist and antagonist of 5-HT2A receptors, has been approved for the treatment of psychosis associated with PD. An open-label phase-two study has indicated that the administration of pimavanserin for 8 weeks could improve depression in PD patients, without impairing motor or cognitive function [59].

Concerning selective norepinephrine reuptake inhibitors (NRIs), reboxetine could exert beneficial effects in PD patients with depression in an open-label study [60]; whereas, the efficacy of atomoxetine in another study among PD patients with depression was insignificant [61].

Cholinergic dysfunction has also been implicated in the neurobiology of PD depression. Cholinergic deficits have been associated with depressive symptoms in patients with PD [62]; the reduction of α4β2- nicotinic acetylcholine receptor binding was primarily detected in the midbrain, putamen, anterior cingulate, and occipital cortex [63]. Donepezil has not been shown to exert significant effects on depression associated with PD [64]. On the other hand, rivastigmine, one more cholinesterase inhibitor used in AD and dementia that is associated with Lewy body diseases, including Parkinson’s disease dementia, was associated with the improvement of depressive symptoms in patients with PD in an open-label trial [65]; this highlighted the need for future randomized controlled trials to clarify its potential antidepressant benefits, especially for patients with coexisting cognitive impairments.

Glutamatergic neurotransmission is implicated in the pathophysiology of depression and patients with depression display higher levels of glutamate in the basal ganglia, as shown in magnetic resonance spectroscopy (MRS) studies [66]. A double-blind pilot clinical trial has shown that memantine, a N-methyl-D-aspartate (NMDA) receptor antagonist, was not effective in alleviating depressive symptoms in PD patients [67]. Interestingly, it has been recently shown that ketamine, another NMDA receptor antagonist, has been associated with improved depressive behavior in rat models of PD [68]; there is currently a clinical trial investigating its role in PD patients with depression (NCT04944017). Till now, the exact role of glutamatergic neurotransmission and NMDA antagonists in PD depression has remained elusive at a clinical level.

An exploratory post hoc analysis of a randomized placebo-controlled clinical trial has also revealed that the weekly subcutaneous administration of exenatide, a glucagon-like peptide-1 (GLP-1) receptor agonist being widely used for type 2 diabetes, could alleviate depression in PD patients [69]. In mouse models of PD, GLP-1 mimetics can protect against alpha-synuclein deposition, neuroinflammation, dopaminergic neuronal loss, and oxidative stress, as well as motor dysfunction [70]. In addition, GLP-1 receptor activation has been recently associated with improved depressive-like behavior in diabetic mice, possibly by suppressing neuroinflammation, mitochondrial dysfunction, and oxidative stress [71]. Therefore, the clinical benefits and cellular mechanisms underlying the antidepressant effects of exenatide, or other molecules implicated in the GLP-1 pathway, deserve further study.

## 3. Non-Pharmacological Treatments for Depression in PD

Although antidepressant medications remain the mainstay of the management of depression in PD, the detrimental effects of polypharmacy; the potential comorbidities, especially in elderly patients; the possible drug interactions; and the limited therapeutic options for pharmacotherapy-resistant cases have paved the way for the investigation of several non-pharmacological interventions [72]. Herein, we discuss the recent evidence on the wide variety of non-pharmacological treatments for alleviating depressive symptoms in PD (Figure 1 and Table 2).

### 3.1. Psychotherapy (Cognitive Behavioral Therapy, Psychodynamic Psychotherapy)

Psychotherapy represents a useful tool for PD depression, especially for patients who have low tolerances or contraindications for antidepressants [12]. CBT aims to promote coping and problem-solving skills and improve the mood of the patients by analyzing and altering their dysfunctional and maladaptive thinking and behavior. Through CBT, patients can recognize their negative or inaccurate ways of thinking and overcome challenging conditions more effectively. CBT consists of several strategies, including psychoeducation, management of anxiety, behavioral activation, restructuring of cognitive processes (e.g., thoughts), emotional regulation, problem-solving methods, and education on sleep hygiene [73]. It is considered that CBT should focus on specific PD-related aspects, including beliefs about illness, dealing with loss of functioning, the scheduling of activities related to motor functioning, preparing for functional disability, and realizing that some activities might be avoided because of dysfunctional beliefs and not an actual disability [35]. Furthermore, individualized adaptations for potential executive dysfunction and targeting specific problematic areas are important [73]. Mechanistically, CBT may activate the orbitofrontal and anterior cingulate cortexes, which are both closely related to emotional regulation [74].

Randomized clinical trials and meta-analyses have demonstrated that CBT is an effective non-pharmacological strategy for PD depression [11,75,76,77], with an even greater effect compared to antidepressant medications [29]. In particular, a recent meta-analysis of CBT in PD showed that an intervention time period of more than 8 weeks was superior to that of less than 8 weeks and non-group therapy was advantageous compared to group therapy [78]. Possible reasons for the smaller efficacy of CBT in groups may include the less targeted and individualized therapeutic process and the reduced distraction of the other patients in non-group sessions [78]. Studies with follow-up periods of 1–6 months have shown that the beneficial impact of CBT is sustained [79]. Interestingly, a meta-analysis including studies with brief psychotherapy has indicated that psychodynamic psychotherapy is more effective than CBT for depression among PD patients. Psychodynamic psychotherapy is a talk therapy focusing on the formation of a therapeutic relationship exploring the early life experiences of the individuals and how these influence their current thoughts and behaviors [80].

Telemedicine allows for the remote provision of healthcare services for patients with neurodegenerative diseases, including PD, thereby overcoming geographical barriers and allowing for better access to specialized medical care [81,82]. Motor fluctuations and lack of sufficient social support may limit the ability of PD patients to attend psychotherapy sessions in-person [35]. In this context, the use of telephone-based and video-based CBT has been shown to be feasible and effective for patients with PD and depression [83,84]. The remote online delivery (telephone- or video-based) of CBT has shown similar efficacy to the offline approach in a recent meta-analysis, highlighting the equal efficacy of both intervention methods [78].

Hence, there is a substantial amount of evidence indicating that CBT, and potentially brief psychodynamic psychotherapeutic approaches, provide significant benefits for PD patients with depression. However, some aspects should also be considered, including affordability issues, inadequate support from caregivers, and the long-term effects of this approach, since the progressive neurodegenerative process might limit the more than 1–6 months maintenance of its therapeutic benefits after the cessation of the treatment [35].

### 3.2. Physical Activity and Exercise

Physical activity is a broad term referring to any movement generated by the skeletal muscles, which leads to the expenditure of energy, and it involves sports, occupational, or household activities [85]. Although the term “physical exercise” is usually used interchangeably, it implies structured, purposeful, and repetitive movements with the aim to maintain or improve physical fitness, such as aerobic exercise, resistance training, treadmill training, and stationary cycling [85]. Physical activity and exercise have shown significant benefits in a wide range of clinical conditions, including depression and PD. Both motor and non-motor symptoms, such as depression, can be improved with physical exercise [85]. The cellular processes that contribute to the beneficial effects of physical exercise on depression are supposed to involve increased levels of neurotrophic factors, such as brain-derived neurotrophic factor (BDNF); beta-endorphins; and neurotransmitters, such as dopamine, serotonin, and norepinephrine [86]. In PD, physical exercise can promote mesolimbic function and induce the release of dopamine in the striatum [87]. In addition, it can inhibit neuroinflammation, mitochondrial impairment, and oxidative stress, as well as regulating synaptogenesis, neuroplasticity, angiogenesis, and the release of neurotrophic factors [88]. It has been proposed that physical exercise may improve depression in PD by promoting hippocampal neurogenesis, serotonin biosynthesis, the activation of the endogenous cannabinoid system, and cerebral blood flow [89].

Several meta-analyses have confirmed that compared to control interventions, physical activity and exercise could significantly reduce depressive symptomatology in PD patients [85,89,90,91]. In particular, general and aerobic exercise, as well as balance training, could ameliorate depressive symptoms of PD patients while stretching exercises did not have significant antidepressant effects [85]. Regarding intensity, both light-to-moderate and moderate-to-vigorous exercises provided significant benefits, as shown by a recent meta-analysis [91]. Combined training interventions displayed significant effects while aerobic exercise alone did not reach a level of statistical significance in the moderator analysis of this study [91], suggesting that exercise type rather than intensity might substantially affect the antidepressant effects of physical exercise. Another recent meta-analysis demonstrated that, regarding disease duration, physical activity provides significant benefits for depression in PD patients for more than 5 years [89]. Furthermore, the duration and frequency of physical activity are also important since a total duration of more than 12 weeks, for at least than four times weekly, and 60–90 sessions provided the most benefits in the subgroup analyses of this work [89].

Regarding other specific types of physical exercise, a recent meta-analysis showed that although resistance training could improve PD depression, it was not superior to other forms of physical activity [92]. However, a subgroup analysis of another meta-analysis showed that resistance training for 60–90 min, more than four times per week for up to 12 weeks, was the most effective exercise type for alleviating depressive symptoms in PD patients with a disease duration of more than 5 years [89]. Therefore, the determination of the duration and frequency of the physical activity interventions, as well as disease duration among the relevant studies, further limits the comparability of their results.

Despite their beneficial effects on postural balance, aquatic exercise programs could not significantly affect depressive symptoms in PD patients, as shown in another recent meta-analysis [93].

Thus, physical exercise can provide significant benefits for patients with PD and coexisting depression; although, the best exercise type for this population needs to be further explored. In addition, given the diverse requirements of each activity type, therapeutic physical exercise protocols need to be tailored to each patient’s needs and general medical conditions, along with their PD stage, cognitive status, and personal preferences.

#### 3.2.1. Mind–Body Exercises (Yoga, Tai Chi and Qigong)

Other forms of exercise combine physical exercise with mind exercises. Mind–body exercises, mainly including yoga, tai chi, and qigong, are a type of moderate-intensity exercise focusing on muscle stretching, and coordination, relaxation, as well as movement and breathing control [94]. They are widely used for a variety of chronic diseases, including PD, and several studies have revealed their beneficial effects on motor manifestations; but, they also aid with depression, anxiety, and other non-motor symptoms [94].

Yoga, which is widely used globally, is a group of physical and mental practices involving different standing and sitting postures, breathing exercises, and meditation techniques [95]. A pilot clinical trial showed that adaptive yoga exercise was effective in combating PD depression [96] and a mindfulness yoga program could improve depression in PD patients in a recent randomized clinical trial [97]. A recent meta-analysis on the effects of yoga in both motor and non-motor manifestations demonstrated that yoga could significantly improve depressive symptoms in PD patients [95]. Yoga has been associated with reduced cortisol levels, enhanced activity of the GABAergic system, and stimulation of the parasympathetic system, thereby ameliorating anxiety and depression [95]. Hence, yoga can be suggested as a complementary and holistic therapeutic approach for patients with PD, especially for those who may not have the ability to follow more intensive or strenuous exercises.

Tai chi and qigong historically combine components of Asian philosophy, traditional Chinese medicine, and martial arts; they integrate physical and cognitive elements, including flexibility, motor control, balance, coordination, focused attention, multi-tasking, and goal-oriented exercises [98]. Tai chi has been associated with improved outcomes in the emotional domain of quality of life compared to combined stretching–strengthening exercises in PD patients [99]; although, there is also evidence showing a lack of significant efficacy in mood compared to routine exercise activity [100]. The different exercise types in the control group are another limitation that may explain these inconsistent findings. A systematic review confirmed the beneficial effects of qigong on depression among PD patients [85].

Mind–body exercises have shown significant effects on improving depressive symptoms in PD patients compared to the control groups in a recent meta-analysis including studies on yoga, tai chi and qigong [94]. Another meta-analysis has also confirmed the antidepressant effects of tai chi and qigong on PD depression [98]. Hence, mind–body exercises could provide additional benefits for PD patients with depression and could be used as a complementary approach for patients interested in and willing to participate in this exercise type.

#### 3.2.2. Dance

Dance is a multidimensional activity that combines physical exercise with music and rhythm, social interaction, and cognitive regulation, entailing the maintenance of visual, auditory, and sensory reactions, together with perception, memory, and expression [101]. Dance is considered to induce a sense of pleasure and improvement of mood by promoting dopamine release from the ventral tegmental area and the ventral striatum, thereby activating the reward system and basal ganglia circuits [13,102].

Despite the positive results of several relative clinical trials, two meta-analyses demonstrated that dance was not significantly effective at alleviating depression in PD [103,104]. Potential reasons for these findings include the large heterogeneity of the relative studies regarding the age of the patients, the PD stage and duration, the short periods of follow-up, small sample sizes in some of the studies, and methodological biases [104]. However, a recent network meta-analysis showed that dance was the best non-pharmacological approach for improving depression [11]. This is in line with another recent network meta-analysis, demonstrating that dance is the most appropriate exercise for depression [105]. Notably, dance has been linked to increased compliance among PD patients compared to participation in other physical exercise programs [104].

Therefore, despite the lack of very strong evidence, dance remains a promising non-pharmacological treatment approach for PD depression in clinical practice. Future studies with larger sample sizes, longer follow-ups, and a higher methodological quality focusing on standardized dance protocols are required in order to confirm its effects on depressive symptoms in PD.

### 3.3. Electroconvulsive Therapy (ECT)

ECT is a well-established treatment approach for psychiatric diseases, including refractory psychosis and depression, with a favorable safety profile in the elderly population [106]. Several case series, several observational studies, and one randomized controlled trial have demonstrated that ECT may be beneficial not only for psychotic and depressive symptoms in PD but also for motor impairment, even in patients without comorbid psychiatric manifestations [106].

Importantly, systematic reviews have shown that ECT can significantly improve severe depressive symptoms in PD patients without affecting, and even improving in some studies, cognitive function [10,106]. However, temporary confusion, disorientation, and delirium have been reported in some cases, occasionally resulting in the cessation of ECT [10]. In this context, pulse width alterations and electrode placement adjustments have been recently proposed to be related to fewer side effects on cognition [106]. Importantly, ECT was shown to be beneficial for pharmacotherapy-resistant psychotic and depressive manifestations in PD [106]. On the other hand, the optimal treatment protocol for the use of ECT in PD depression (duration, number of sessions, necessity of a tapering course) still remains unclear. Nevertheless, before the initiation of ECT sessions, a detailed medical evaluation is needed, including cardiac history, medications, and consent obtainment, in collaboration with the family members of the patient [12].

Although the mechanism underlying the beneficial effects of ECT in depression has not been elucidated yet, it has been suggested to involve neuroplastic modulation in the hippocampal area [106]. In rat models of PD, ECT can promote dopamine D1 and D3 receptor binding in the striatum [107]; patients with PD display higher homovanillic acid levels in the cerebrospinal fluid after ECT therapy [108]. Hence, it seems that dopaminergic regulation may contribute to the antiparkinsonian effects of ECT while further evidence is needed to elucidate its specific effects on PD depression.

### 3.4. Deep Brain Stimulation (DBS)

DBS in the subthalamic nucleus (STN), which is successfully used for motor symptoms, may also exert beneficial effects for depressive symptoms and impulse control disorders in PD [109]. In particular, stimulation in the ventral, anterior, and medial sites of the STN connected with limbic areas is linked to a more pronounced impact on non-motor symptoms [110]; meanwhile, pallidal and thalamic stimulation is considered rather neutral for depression [6]. On the other hand, a higher risk for postoperative suicide behavior has been shown to be possibly related to the DBS of the STN compared to the general population [111]; although, there is also evidence not supporting this observation [112].

Recently, a meta-analysis indicated that, postoperatively, DBS in the STN is associated with improved anxiety and depressive symptoms in PD patients [113]. In addition, despite the described reports of mania after DBS, no significant alterations in manic symptomatology were identified in this study [113]. However, another meta-analysis that aimed to compare various neuropsychological and neuropsychiatric outcomes after DBS in the STN, compared to that in the globuspallidus internus (GPi) in patients with PD, demonstrated no significant differences regarding depressive symptoms [114]. In agreement with this evidence, compared to that of the GPi, DBS in the STN caused similar effects on depressive symptomatology after the intervention [115]. Post-surgery, depressive symptoms were shown to be reduced after DBS in the STN and GPi in another meta-analysis [116]. In summary, it seems that DBS in the STN might provide some benefits for depressive symptoms in PD; although, the current evidence is too inconsistent to draw definite conclusions.

### 3.5. Non-Invasive Brain Stimulation (NIBS)

Non-invasive brain stimulation (NIBS) utilizes weak currents or magnetic pulses in order to alter neurophysiological activity in the brain that is considered to be related to clinical conditions. The m frequently used forms of NIBS include rTMS and transcranial direct current stimulation (tDCS) [117]. rTMS creates a focal and strong pulsed electromagnetic field via a coil over the patients’ scalp, thereby depolarizing the neurons underneath [118]. The rTMS application may also affect the activity of various molecules and regulate several cellular processes, including oxidative stress, neurotransmission, neuroplasticity, gene expression, cell apoptosis, and neuroinflammation [117]. On the other hand, via tDCS, the weak current is applied through scalp electrodes, resulting in the depolarization of neurons, modulation of neuronal excitability, and alteration of cerebral blood flow [117].

Several studies have demonstrated that rTMS provides significant benefits for PD patients with depression [119,120]; although, there is also evidence not confirming these findings [121]. These contradictory results might be attributed to methodological discrepancies, including the different baseline characteristics of the patients regarding age, the stage and duration of the disease, the simultaneous use of antidepressants, and the type and method of the control group (sham stimulation, clinical monitoring, or other), as well as the different protocol used for the rTMS application [76]. Meta-analyses have revealed that rTMS in the prefrontal cortex can indeed alleviate depressive symptoms in PD patients [76,117,122,123,124,125,126]. However, high-frequency rTMS was not superior compared to sham rTMS or SSRIs [127], or a placebo [128], for depression in PD. Importantly, studies that compared the effectiveness of rTMS with that of antidepressants did not show significant differences [76,125]. However, it has also been shown that rTMS in combination with antidepressant treatment was more effective for PD depression than the use of antidepressants alone [117]. Concerning safety, rTMS was well-tolerated and no serious adverse effects were reported; while transient, mild headache and neck pain were the most frequent complications [76]. The different statistical methods and inclusion criteria may account for the partially conflicting results of the abovementioned studies.

Depression has been associated with reduced regional cerebral blood flow (rCBF) in the prefrontal cortex of patients with PD [129]; rCBF was found to be higher after rTMS treatment in depressed PD patients [130]. This evidence suggests that the restoration of hypoperfusion in the prefrontal cortex may underly the antidepressant effects of rTMS in PD depression. Notably, the antidepressant effect of rTMS has been proposed to be mediated by an intact functional connection between the cortex, close to the site of the stimulation, and deeper brain areas [131]. Therefore, it could be speculated that the effectiveness of rTMS against depression might be diminished during the course of neurodegeneration at more advanced stages of PD due to the greater and more widespread damage of involved pathways. This mechanism might also partially explain the conflicting results of some relative studies that included patients at different stages of the disease.

Regarding tDCS, the number of studies that have investigated its utility in PD is much fewer compared to the number of those investigating rTMS and sample sizes are small. A recent meta-analysis was inconclusive about the effectiveness of tDCS in PD depression due to insufficient evidence [117].

Collectively, despite the lack of strong evidence, rTMS seems to be a possibly useful complementary treatment option for PD patients suffering from depression. However, it would be important to elucidate the patients’ characteristics that may facilitate its antidepressant effects via sufficient randomization, larger sample sizes, and subgroup analyses of future studies.

### 3.6. Bright Light Therapy (BLT)

Bright light therapy (BLT) is effective for sleep disorders associated with circadian rhythm disturbances and seasonal affective disorder; it does so by modulating circadian rhythm through melatonin [132,133]. Retinal exposure to light suppresses melatonin release from the pineal gland, which inhibits sleep. Light may promote alertness by inhibiting melatonin, stimulating the monoaminergic system that is related to arousal, and suppressing the activity of the ventrolateral preoptic nucleus, which induces sleep [132]. Furthermore, light can reduce the levels of serotonin reuptake transporters and elevate that of serotonin in the brain regions implicated in emotional modulation [134].

A randomized controlled trial has indicated that BLT can exert beneficial effects on PD patients with depression; although, it was not superior enough to control light [135]. However, BLT was more effective in ameliorating depressive symptoms in PD compared to the placebo group in another study [136]. A recent review and meta-analysis found that although among the open-label non-controlled clinical studies BLT could effectively improve depression in PD patients, no significant effects were identified in the following meta-analysis of randomized controlled trials [132]. The authors of this work proposed that the relatively small sample sizes of the included studies and the inconsistent protocols of BLT application, including diverse doses, timings, and durations of treatment, might explain the lack of statistical significance [132]. On the contrary, another more recent meta-analysis indicated that light therapy, including BLT and blue–green light therapy, exerted beneficial effects on PD depression compared to the control groups [137]. Future studies with more standardized treatment BLT protocols are needed to clarify its effects on PD depression in clinical practice.

### 3.7. Acupuncture

The broader term of acupuncture refers to the stimulation of specific body sites with needles, electricity, lasers, or pressure [138]. Acupuncture has been widely used for various psychiatric and neurological disorders, including motor and non-motor symptoms of PD. A meta-analysis demonstrated that acupuncture-related therapies showed inefficacy in improving depressive symptoms in PD patients [138]; another one showed that it was effective in alleviating depression in PD [139] and a 2-month course was found to represent the optimal intervention duration [139]. The different inclusion criteria and the broader definition of acupuncture therapies in the first meta-analysis may explain the different findings. Mechanistically, it has been recently revealed that acupuncture may alleviate depressive behavior in rat models of PD, possibly by upregulating the mTOR pathway, suppressing autophagy, increasing dopamine levels, reducing alpha-synuclein content, and enhancing the expression of synaptic proteins [140]. Hence, acupuncture may represent an effective complementary treatment approach for PD depression; although, the optimal methods and techniques for this population need further investigation.

### 3.8. Lee Silverman Voice Treatment (LSVT)

The Lee Silverman Voice Treatment (LSVT) is an exercise-based behavioral therapy program that involves amplitude training combined with sustained attention of a sole movement, thereby enabling long-term maintained physical and cognitive participation in functional activities [141]. There are mainly two types of LSVT: the LSVT-LOUD, which focuses on speech, and the LSVT-BIG, which focuses on the limb motor system [141]. Interestingly, a clinical trial indicated that the LSVT-BIG could improve depressive symptoms in PD patients compared to general exercise [142]; although, there is also evidence not replicating these effects [143]. However, a recent meta-analysis showed that the LSVT-BIG was the second most effective non-pharmacological intervention for PD depression after dance intervention [11], suggesting that the LSVT holds a promising potential and its efficacy in PD depression needs to be further examined.

### 3.9. Other Non-Pharmacological Treatments (Music Therapy, Active Theater, Massage Therapy, Cognitive Training, Virtual Reality)

There is also a variety of other non-pharmacological treatments, such as music; music-contingent gait training via a technique called Ambulosono, which utilizes step size for controlling the real-time playing of motivational music, could reduce anxiety and depressive symptoms in patients with PD [144]. However, no significant effects on depressive symptoms were detected in patients with PD as a result of group music therapy with voice and singing intervention in another study [145].

Another example is active theater. A randomized controlled single-blinded study indicated that participation in active theater for 3 years could significantly improve depression in PD patients compared to the control group receiving physiotherapy [146]. Another study demonstrated that psychodrama with role-playing in groups significantly alleviated depressive symptoms in PD patients compared to the control group that received no intervention [147]. Social interaction, combined with the necessity of controlling body movements in order to impersonate a theater character, has been proposed as a potential mechanism [146].

Regarding massage therapy, a recent systematic review indicated that several non-motor symptoms in PD patients, including depression, were improved via traditional Japanese, Thai, Yin Tui Na, and classical deep therapeutic massage [148]; the neurobiological processes that may contribute to these effects remain largely unknown.

Cognitive training incorporates the structured teaching of various strategies or guided practices on particular tasks focusing on specific cognitive domains [149]. Cognitive training has shown beneficial effects on cognitive function in patients with mild cognitive impairments and healthy-aged individuals [149]. In PD, a meta-analysis of randomized controlled trials indicated that cognitive training could indeed improve cognitive function; but, the results were statistically insignificant for depression [149].

More recently, rehabilitation methods have been expanded by the use of virtual reality, which represents an innovative technological method, via which the simultaneously auditory, visual, and tactile stimulation can promote cognitive and motor function [150]. Rehabilitation training based on virtual reality has gained increasing attention for neurodegenerative diseases, including PD. A recent systematic review and meta-analysis concluded that virtual reality rehabilitation training could improve depressive symptoms in PD compared to control groups; video game consoles were shown to enhance the beneficial effects [150]. Virtual reality dance exercises could alleviate depressive symptoms in PD patients compared to controls [151], suggesting that virtual reality holds a promising potential for aiding in the management of PD depression.

## 4. Discussion and Future Perspectives

In summary, although the results of the abovementioned clinical studies and meta-analyses are rather conflicting, current recommendations suggest the use of SSRIs and SNRIs as a first-line pharmacological approach for managing PD depression; whereas, several non-pharmacological treatments represent a useful additional tool for addressing depression in PD, acting more often as an adjunctive strategy (e.g., CBT or physical exercise) or a possible solution for refractory cases (e.g., ECT). Depression necessitates a holistic therapeutic approach in PD. However, the selection of the most appropriate treatment depends on several factors, including age, the stage of the PD, the severity of the depression, the patients’ clinical profile, existing comorbidities, potential medications’ side effects, patients’ personal preferences, the availability of each service in each geographical region, and socioeconomic conditions.

In early PD, younger age is associated with a higher likelihood of the resolution of depression, especially in mild cases [6,152]; although, the factors that affect the course of depression at an individual level need further investigation. The stage of PD majorly affects the available treatment options for PD depression. Several studies and meta-analyses have included PD patients at Hoehn and Yahr Stages 1–2 or 3 [11]; the effects of therapeutic approaches on depression at later PD stages are still obscure. More advanced stages and longer durations of PD have been associated with depression and longer durations have also been related to a reduced probability of a resolution of the depressive symptoms [6,152,153]. Therefore, it would be useful for future studies to consider age, disease stage, and duration as factors possibly affecting the efficacy of pharmacological and non-pharmacological treatments; subsequently, the optimal therapeutic choices for PD depression should be based on these parameters.

Importantly, PD is a clinically heterogeneous disorder with various endophenotypes that may affect the treatment choices for motor and non-motor symptoms. Most clinical trials investigating the effectiveness of pharmacological treatments in PD have excluded patients with co-existing dementia [12]. Cognitive impairment may limit the efficacy of several non-pharmacological interventions for depression, including CBT, active theater, occupational therapy, dance, or physical exercise programs [11]. In fact, depressive symptoms are associated with dementia in PD and, as the disease progresses, cognitive decline deteriorates depression. Furthermore, clinical trials based on CBT and other non-pharmacological treatments usually exclude PD patients with dementia [11]. Accumulating evidence supports that dementia-related depression is a rather distinct clinical entity from that observed among PD patients without dementia, possibly reflecting a more extensive neurodegenerative burden [6]. Although the effects of subtle deficits, such as mild cognitive impairment, on the efficacy of CBT or other interventions remain unclear, the management plan for each PD patient with depression and dementia should be individualized and tailored to the patient’s personal needs.

The severity of depression has been associated with an akinetic-rigid phenotype, bradykinesia, and axial symptoms in PD, possibly reflecting more pronounced dopaminergic deficits [6]. In agreement with this evidence, dopaminergic metabolism has been shown to be bilaterally reduced in the caudate nucleus and putamen of PD patients and correlated with the severity of depressive symptoms [154]. Interestingly, it has also been shown that the magnitude of the flexion of the lower trunk of PD patients is associated with depression severity [155]. Hence, it could be speculated that more severe forms of depression may require interventions targeting dopaminergic pathways, such as the possibly additional use of dopaminergic agonists.

Long-term dopaminergic therapy is usually associated with the development of levodopa-induced dyskinesias and motor fluctuations, which have been associated with depression in PD [156]. Most clinical trials of pharmacological treatments have excluded PD patients with co-existing severe levodopa-induced motor fluctuations [12], raising concerns about the generalizability of their findings for patients with levodopa-induced motor complications. Future studies should also include a subgroup of patients with advanced PD and coexisting motor complications, in order to clarify the optimal therapeutic strategies for this subpopulation.

Concerning genetic forms of PD, it has been demonstrated that PD patients with Parkin mutations display an increased prevalence of depressive symptoms compared to PD patients without known causative gene mutations [157]. Two systematic reviews have shown that PD patients carrying GBA1 mutations exhibit depression more often compared to non-carriers [158,159]. Furthermore, despite therapeutic intervention, PD patients with LRRK2 gene mutations and mood disorders have considerably high depression and anxiety rates. PD patients with mood-related disorders experience high rates of anxiety and depression [160]. In this context, clinical vigilance for depression screening for patients known to carry these mutations is needed. In addition, it would be important to clarify if future gene-targeted therapeutic approaches would be effective for both motor and non-motor PD-related symptoms.

Greater α-synuclein deposition, but not hyperphosphorylated tau- or beta-amyloid pathology, has been correlated with depression in the SNpc, nucleus accumbens, and ventral tegmental area of patients with Lewy body disorders (PD with and without dementia, and dementia with Lewy bodies) [161]. Therefore, although available results from clinical trials using immunotherapeutic approaches targeting alpha-synuclein are not positive [162,163], it might be expected that the development of future effective pharmaceutical drugs targeting alpha-synuclein may exhibit beneficial effects on non-motor symptoms too, including depression.

Accumulating evidence highlights the role of neuroinflammation in the pathophysiology of PD and PD depression; in PD, depression has been related to increased levels of the pro-inflammatory molecules tumor necrosis factor-alpha (TNF-α), soluble interleukin-2 receptor (sIL-2R), and C-reactive protein (CRP) [6,164]. A recent meta-analysis has demonstrated that anti-inflammatory agents, including non-steroidal anti-inflammatory drugs, statins, omega-3 fatty acids, and minocyclines had significant effects on major depressive disorder [165]. In addition, pioglitazone, glucocorticoids, and cytokine inhibitors have also shown promising results [166]. The administration of fish oil, which contains omega-3 fatty acids, has been shown to be effective in improving depressive symptoms in PD patients in a small placebo-controlled pilot study [167]. Although omega-3 fatty acids have pleiotropic mechanisms of action [168], it can be speculated that other anti-inflammatory agents may exert beneficial effects against PD depression as well.

The gut–brain axis also plays an important role in PD and depression pathophysiology [169]. In comparison with non-PD individuals, there is a disequilibrium between the bacteria contributing to a pro-inflammatory versus anti-inflammatory microenvironment in patients with PD [169]. Gut bacteria are also able to produce several neurotransmitters, including serotonin, norepinephrine, dopamine, and acetylcholine [169]. Probiotics, such as *Bifidobacillus* and *Lactobacillus*, can restore epithelial integrity and regulate inflammation [169]. Two recent meta-analyses have shown that oral probiotics can improve not only motor but also several non-motor symptoms in PD, including depression [170,171]. Furthermore, there is also an ongoing clinical trial aiming to identify the effects of probiotic treatment on depression for patients with PD (NCT05568498).

Edible mushrooms contain precursors of serotonin; psilocybin is a natural psychedelic compound produced by various fungi species, which has shown beneficial effects for depression via anti-oxidant and anti-inflammatory effects, as well as by regulating neurogenesis, the gut–brain axis, and the expression of neurotrophic factors [172]. Interestingly, a clinical trial is currently investigating its role in depression for patients with PD (NCT04932434).

A higher white matter burden may be associated with depression in the elderly population and white matter lesions are considered to contribute to PD depression as well [173]. PD patients suffering from depression display impaired integrity of white matter long fibers in their left-brain hemisphere [174]. Hence, cardiovascular risk factors should be appropriately screened and treated in patients with PD, especially those with depression.

Regarding the limitations of the relevant studies discussed above, most clinical trials have investigated the short-term effects of both pharmacological and non-pharmacological treatments on PD depression; however, their long-term effectiveness remains largely unknown. Furthermore, studies examining the effects of non-pharmacological interventions on PD depression are characterized by substantial heterogeneity, regarding the sample sizes, the eligibility criteria, the duration, the frequency of the intervention used, and the assessment methods and scales for depressive symptoms, as well as the severity of depression and/or PD stage. These factors may affect the effectiveness of both pharmacological and non-pharmacological approaches. In addition, there is a large variability regarding the diagnosis of depression; some studies have included patients with a depressive disorder according to the Diagnostic and Statistical Manual of Mental Disorders 3rd revised, 4th, or 5th edition (DSM-III-R, DSM-IV or DSM-V) diagnostic criteria (major depressive disorder, minor depressive disorder, or dysthymic disorder); but, others have used the cut-off points of depression scales in order to define the presence of depressive symptoms. Some studies have only included patients with major depression; although, it has been suggested that the diagnostic criteria for major depression might not be appropriate for patients with neurodegenerative disorders. One important limitation of the use of DSM criteria is the fact that some manifestations of depression, including psychomotor retardation and loss of energy, are commonly present in PD. Depression is affected by biopsychosocial factors while the studies of therapeutic approaches do not usually take into account psychosocial aspects, such as personality traits. This important limitation applies to PD depression as well. Furthermore, psychotherapy and pharmacotherapy are distinct in nature and the efficacy of psychotherapy depends on several factors. Randomized controlled trials do not adequately assess the relationship between therapists and patients, which may largely affect the effectiveness of the psychotherapeutic process. Future studies should also consider this factor when evaluating the effects of psychotherapy on PD depression. Larger studies with longer follow-up periods, involving head-to-head comparisons or well-selected placebo-controlled groups that would also ideally allow subgroup analyses, will aid in the identification of the most appropriate and individualized treatment approaches.

In this comprehensive review, we aimed to provide an updated overview of the literature regarding the various pharmacological and non-pharmacological approaches to PD depression and discuss the main findings and potential future perspectives. We have not conducted a systematic review or meta-analysis of the relevant studies; however, we followed a systematic approach for the literature search and we believe that we have included all key relevant studies.

## 5. Conclusions

Collectively, no optimal treatment for PD depression can be currently recommended; the therapeutic approach should be individualized according to patients’ comorbidities, medications and general health status, depression severity, and the stage of their PD, as well as their socioeconomic conditions. As a general recommendation, pharmacological therapy is usually first suggested; non-pharmacological interventions could be additionally used or possibly proposed as even a first choice for patients with mild symptoms. Future, larger well-designed clinical trials for both pharmacological and non-pharmacological interventions are needed in order to clarify the most appropriate therapeutic strategies for the holistic and personalized management of PD patients with depression.

## Figures and Tables

**Table 1:** Main types and examples of pharmacological and non-pharmacological treatments for depression in Parkinson’s disease.

Pharmacological Treatments	
Type	Examples
SSRIs	citalopram, sertraline, paroxetine, etc.
SNRIs	venlafaxine, duloxetine, etc.
TCAs	desipramine, amitriptyline, nortriptyline, etc.
Levodopa	
Dopaminergic agonists	pramipexole, ropinirole, rotigotine, piribedil, apomorphine
MAO-B inhibitors	selegiline, rasagiline, safinamide
Others	5-HTP, trazodone, nefazodone, vortioxetine, pimavanserin, reboxetine, rivastigmine, ketamine, exanetide

**Table 2:** Main types and examples of non-pharmacological treatments for depression in Parkinson’s disease.

Non-Pharmacological Treatments	
Type	Examples
Physical activity and exercise	aerobic exercise, resistance training, balance training, etc., dance, mind–body exercises (tai chi, qigong, yoga)
ECT	
NIBS	rTMS, tDCS
Acupuncture	
LSVT	
DBS	
Bright light therapy	
Others	music therapy, active theater, cognitive training, virtual reality

## Data Availability

Not applicable.
